# Feasibility and safety of non-contrast optical coherence tomography imaging using hydroxyethyl starch in coronary arteries

**DOI:** 10.1038/s41598-023-40363-7

**Published:** 2023-08-24

**Authors:** Dong Oh Kang, Hyeong Soo Nam, Sunwon Kim, Hongki Yoo, Jin Won Kim

**Affiliations:** 1 Multimodal Imaging and Theranostic Laboratory, Cardiovascular Center, Korea University Guro Hospital, 148 Gurodong-Ro, Guro-Gu, Seoul, 08308 Republic of Korea; 2 Cardiovascular Center, Korea University Guro Hospital, 148 Gurodong-Ro, Guro-Gu, Seoul, 08308, Republic of Korea; 3 Department of Mechanical Engineering, Korea Advanced Institute of Science and Technology, 291 Daehak-Ro, Yuseong-Gu, Daejeon, 34141 Republic of Korea; 4 Cardiovascular Center, Korea University Ansan Hospital, 123 Jeokgeum-Ro, Danwon-Gu, Ansan, 15355, Republic of Korea

**Keywords:** Interventional cardiology, Cardiology, Cardiovascular diseases, Coronary artery disease and stable angina

## Abstract

Intracoronary optical coherence tomography (OCT) requires injection of flushing media for image acquisition. Alternative flushing media needs to be investigated to reduce the risk of contrast-induced renal dysfunction. We investigated the feasibility and safety of pentastarch (hydroxyethyl starch) for clinical OCT imaging. We prospectively enrolled 43 patients with 70 coronary lesions (46-stented; 24-native). Total 81 OCT pullback pairs were obtained by manual injection of iodine contrast, followed by pentastarch. Each pullback was assessed frame-by-frame using an automated customized lumen contour/stent strut segmentation algorithm. Paired images were compared for the clear image segments (CIS), blood-flushing capability, and quantitative morphometric measurements. Overall image quality, as assessed by the proportion of CIS, was comparable between the contrast- and pentastarch-flushed images (97.1% vs. 96.5%; p = 0.160). The pixel-based blood-flushing capability was similar between the groups (0.951 [0.947–0.953] vs. 0.950 [0.948–0.952], p = 0.125). Quantitative two- and three-dimensional morphometric measurements of the paired images correlated well (p < 0.001) with excellent inter-measurement variability. All patients safely underwent OCT imaging using pentastarch without resulting in clinically relevant complications or renal deterioration. Non-contrast OCT imaging using pentastarch is clinically safe and technically feasible with excellent image quality and could be a promising alternative strategy for patients at high risk of renal impairment.

## Introduction

Intravascular optical coherence tomography (OCT) is a high-resolution imaging modality that enables microstructural characterization of the coronary vasculature and peri-procedural adverse findings^1,2^. Following recent technical advances, the use of intravascular OCT has been widely increased in catheterization laboratories over the last two decades. However, contemporary intracoronary OCT has an inherent limitation that it requires the injection of flushing media for image acquisition to avoid signal attenuation by red blood cells^3^. Currently, iodine contrast is the most commonly used flushing solution for OCT imaging; however, greater amount of contrast media by repetitive pullbacks may increase the risk of contrast-induced nephropathy (CIN), especially in high-risk groups^4,5^. Among the various patient- and procedure-related factors, contrast volume has been considered as a modifiable factor that can be reduced to minimize developing CIN^6,7^. Therefore, there is a growing need for alternative flushing media, other than iodine contrast, to reduce the risk of renal dysfunction.

Flushing media for intravascular OCT must be biologically safe and have sufficient viscosity to ensure clear image acquisition^8^. Pentastarch is a synthetic hydroxyethyl starch (HES) solution that was originally developed as a plasma volume expander and has been used in acute care medicine for resuscitation fluid therapy^9–11^. Generally, the administration of small doses of HES solution is reported to be safe without resulting in clinically meaningful adverse events^10,12,13^. Pentastarch has an average viscosity of 4.25 centipoises (cP) at body temperature (37 °C), which is greater than that of blood (3.5 cP at 37 °C)^14^. Given its clinical availability and rheological characteristics, pentastarch could be suggested as an alternative flushing medium for non-contrast OCT imaging. However, no previous studies have examined its feasibility and safety for intravascular OCT. In this study, we hypothesized that pentastarch is highly feasible and clinically safe for intravascular OCT imaging. To answer these questions, here, we prospectively compared the overall image quality and blood-flushing capability with the paired OCT images of iodine contrast and pentastarch using our customized pixel-based comparison algorithm. Additionally, we serially assessed the temporal changes in key laboratory profiles to validate the clinical safety of pentastarch.

## Results

### Baseline characteristics and procedural findings

**Figure 1:**
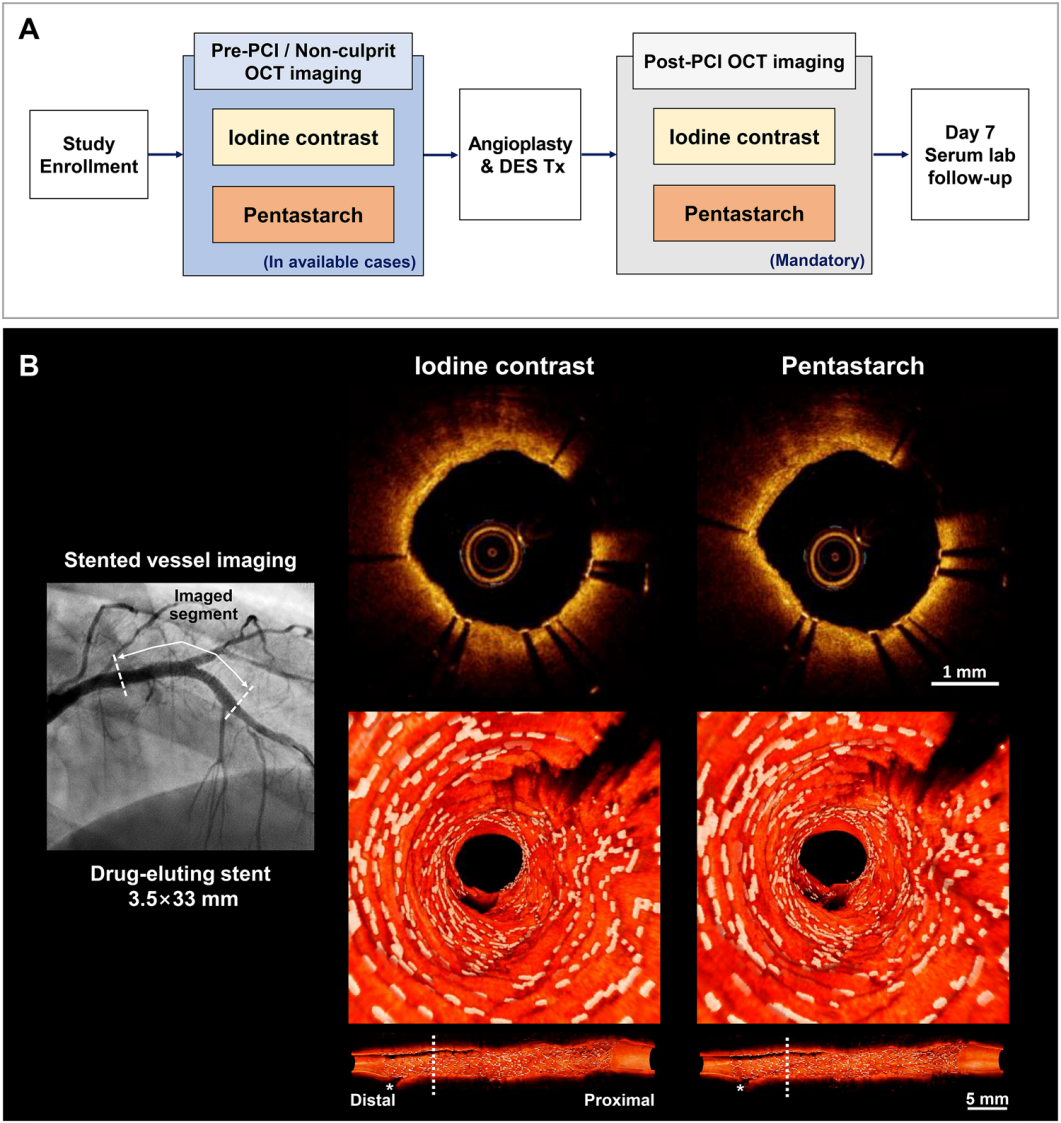
Schematic diagram of the study flow chart. (**A**) This study enrolled patients who underwent OCT-guided PCI for coronary artery disease. Pre-PCI and non-culprit vessel imaging were performed in the available cases, and all stented lesions were imaged after PCI for stent optimization. (**B**) A coronary angiogram obtained after stenting the proximal lesion of the left anterior descending artery (left). Cross-sectional OCT images obtained from a stented vessel using iodine contrast and pentastarch (right upper panel) and customized 3D-rendered reconstruction of paired OCT images (right lower panel). *3D* three-dimensional, *DES* drug-eluting stent, *OCT* optimal coherence tomography, *PCI* percutaneous coronary intervention.

The schematic diagram of the study flow chart is shown in Fig. 1. A total of 81 OCT pullback pairs from 43 patients, 54 arteries, and 70 lesions were prospectively enrolled. The baseline demographic characteristics and procedural findings are shown in Table 1. A total of 20 (46.5%) patients presented with acute coronary syndrome (ACS), and 23 (53.5%) patients were diagnosed with chronic stable angina (CSA) at admission. Laboratory findings showed that 10 (23.3%) and 14 (32.6%) patients had chronic kidney disease (CKD) and heart failure, respectively, at baseline. About half of the patients (n = 22; 51.1%) underwent intracoronary OCT and interventional procedure using 7-Fr guiding catheter, more frequently in those who had worse cardiac and renal functions at baseline (Supplementary Table S1). The following vessels were imaged during the procedure: left anterior descending artery (LAD; 37/54, 68.5%), left circumflex artery (LCX; 9/54, 16.6%), and right coronary artery (RCA; 8/54, 14.9%). Among the 81 OCT pullback pairs, 25 (30.9%) were obtained from native vessels during pre-percutaneous coronary intervention (PCI) or non-culprit vessel imaging, and the remaining 56 (69.1%) pairs were imaged from stented lesions.

**Table 1:** Baseline demographic and procedural characteristics.

Baseline demographic features	
Sex (male)	31 (72.1)
Age (years)	65.5 ± 9.7
BMI (kg/m^2^)	25.3 ± 3.2
Clinical presentation	
Stable angina	23 (53.5)
Acute coronary syndrome	20 (46.5)
Hypertension	28 (65.1)
Diabetes	16 (37.2)
Dyslipidemia	29 (67.4)
Chronic kidney disease	10 (23.3)
Heart failure	14 (32.6)

Laboratory findings	
Serum creatinine (mg/dL)	0.87 (0.72–1.12)
Cystatin C (mg/dL)	0.90 (0.72–1.18)
Estimated GFR (mL/min)	83.0 (58.5–100.0)
AST (IU/L)	25.0 (19.0–38.0)
ALT (IU/L)	24.0 (17.0–39.0)
Total bilirubin (mg/dL)	0.65 (0.48–0.85)
LDL cholesterol (mg/dL)	95.0 (68.0–126.0)
hs-CRP (mg/L)	2.05 (0.63–11.38)
HbA1c (% of Hgb)	5.8 (5.6–7.3)
NT-proBNP (pg/mL)	208.0 (62.5–1389.0)
LVEF (%)	60.0 (48.0–60.0)

Procedural characteristics	
Guiding catheter	
6-Fr catheter	21 (48.9)
7-Fr catheter	22 (51.1)
Average stent number (per patient)	1.19 ± 0.63
Average stent diameter (mm)	2.95 ± 0.39
Total stent length (mm)	31.05 ± 16.74
Fluoroscopic time (min)	18.8 ± 8.5
Radiation exposure (mGy)	1330.5 ± 692.4
Total iodine contrast (mL)	206.9 ± 66.0
Enrolled vessels (n = 54)	
LAD	37 (68.5)
LCX	9 (16.6)
RCA	8 (14.9)
Average number of imaged vessels (per patient)	1.26
OCT pullback pairs (n = 81)	
Stented lesion (post-PCI)	56 (69.1)
Native vessel (pre-PCI)	14 (17.3)
Native vessel (non-culprit)	11 (13.6)
Average number of pullback pairs (per patient)	1.88

Data are expressed as n (%), mean ± standard deviation or median (interquartile range). *ALT* alanine transaminase, *AST* aspartate transaminase, *BMI* body mass index, *Fr* French, *eGFR* estimated glomerular filtration rate, *Gy* gray, *HbA1c* glycated hemoglobin, *hs-CRP* high-sensitivity C-reactive protein, *LAD* left anterior descending artery, *LDL* low-density lipoprotein, *LCX* left circumflex artery, *LVEF* left ventricular ejection fraction, *NT-proBNP* N-terminal pro-B-type natriuretic peptide, *OCT* optical coherence tomography, *PCI* percutaneous coronary intervention, *RCA* right coronary artery.

### Pixel-based image segment analysis and comparison of overall image quality

**Figure 2:**
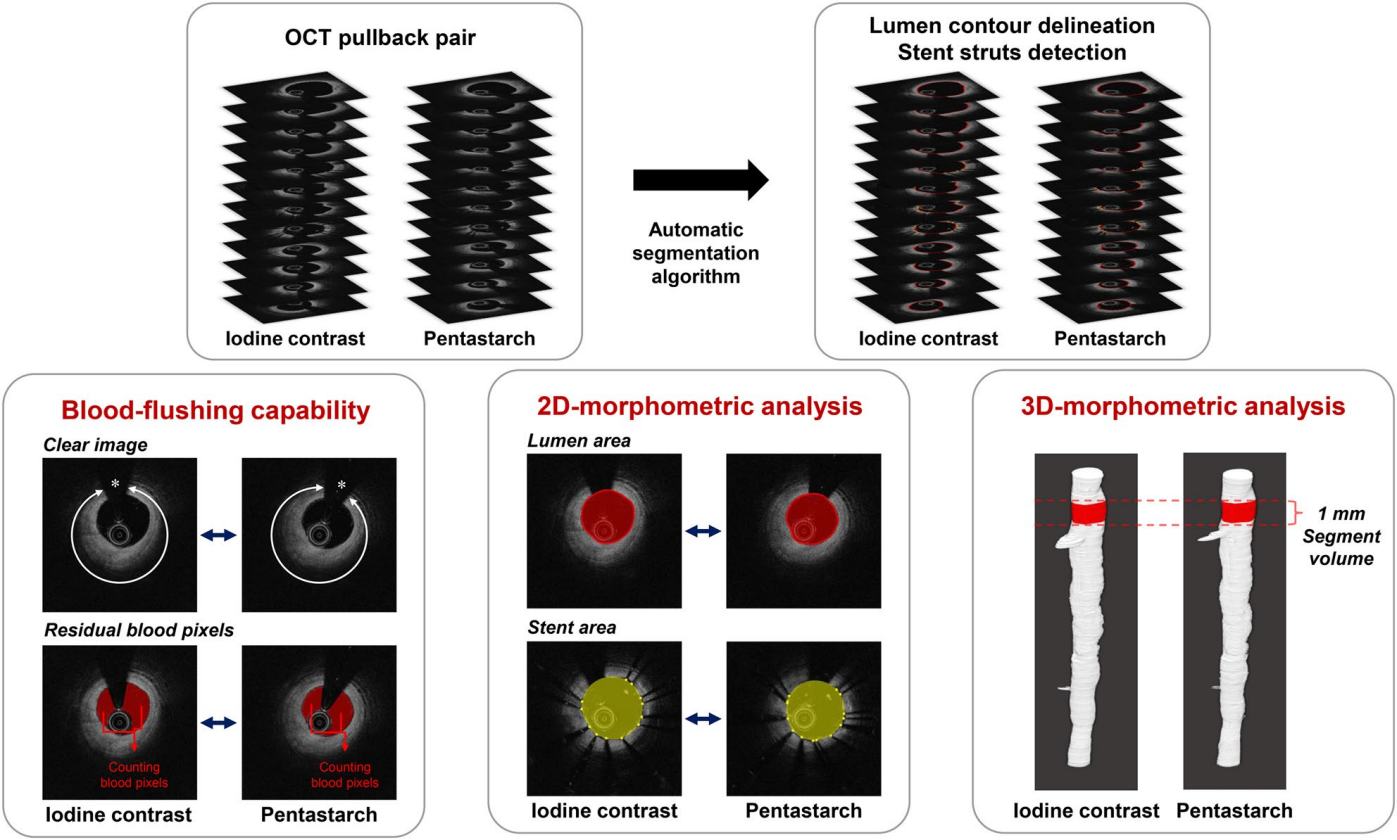
Automated customized pixel-based comparison algorithm for the paired OCT images. An automated customized lumen contour and stent strut segmentation algorithm was adopted for the quantitative comparison of OCT-derived parameters between the paired images. The arterial lumen and stented vessel wall were accurately separated, and residual blood pixels within the vessel lumen were robustly assessed to determine blood-flushing capability. The lumen and stent areas were sampled automatically from all cross-sectional OCT images using the segmentation results and further integrated to provide longitudinal segment volumes. *2D* two-dimensional, *3D* three-dimensional, *OCT* optical coherence tomography.

**Figure 3:**
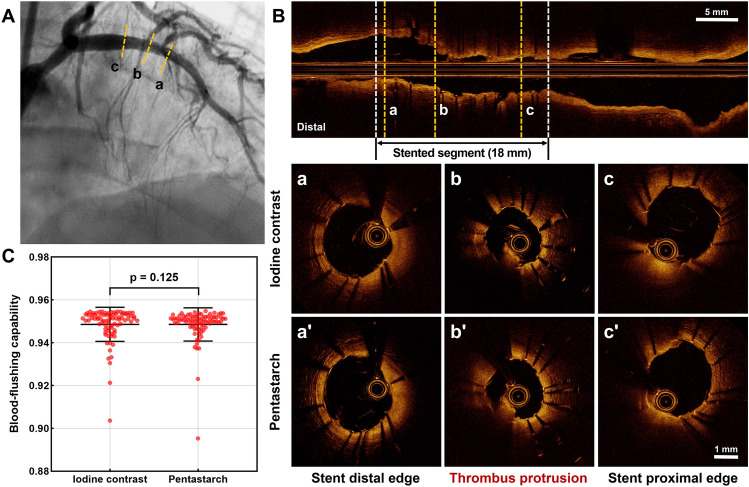
Assessment of overall image quality and blood-flushing capability. A coronary angiogram obtained after stenting the proximal lesion of the left anterior descending artery (**A**). The overall image quality (**B**) and pixel-based blood-flushing capability (**C**) of the paired OCT images were compared between iodine contrast and pentastarch. *OCT* optical coherence tomography.

Figure 2 shows the overall scheme of the OCT image analysis conducted by an automated customized pixel-based comparison algorithm. Results of the pixel-based image segment analysis are presented in Table 2. The number of OCT pullbacks classified as clear runs showed no significant difference between iodine contrast versus pentastarch (91.4% vs. 90.1%; p = 0.786). The overall image quality was comparable between images obtained using iodine contrast and pentastarch (Fig. 3A,B). A total of 5727 1-mm longitudinal segments (2875 obtained using iodine contrast and 2852 obtained using pentastarch) were included in the analysis. The number of clear image segment (CIS) was 2792 (97.1%) and 2751 (96.5%) in the iodine contrast and pentastarch groups, respectively (p = 0.160). The distribution of CIS per pullback showed no significant difference between iodine contrast versus pentastach (p = 0.384; Supplementary Fig. S1). Quantitative assessment of the blood-flushing capability showed no significant difference between the paired OCT pullbacks (median [IQR] 0.951 [0.947–0.953] vs. 0.950 [0.948–0.952]; p = 0.125; Fig. 3C). Average number of pullback attempts for image acquisition was similar between the groups (2.12 ± 1.01 vs. 2.42 ± 1.18, p = 0.204). For blood clearance during image acquisition, relatively larger volume of flushing media was injected with pentastarch compared to iodine contrast (11.19 ± 0.77 vs. 14.84 ± 0.97 mL; p < 0.001). During OCT image acquisition using pentastarch, we observed that a lower volume was needed when using a 6-Fr compared to a 7-Fr guiding catheter (14.33 ± 0.66 vs. 15.32 ± 0.99 mL per pullback, p < 0.001; Supplementary Table S1). However, the pixel-based blood-flushing capability showed no significant difference between OCT images obtained using 6-Fr and 7-Fr guiding catheters with pentastarch flushing (0.950 [0.949–0.951] vs. 0.951 [0.948–0.952], p = 0.663; Supplementary Table S1).

**Table 2:** Overall image quality and pixel-based image segment analysis.

	Iodine contrast	Pentastarch	p-value
Total OCT runs			
Clear runs	74 (91.4)	73 (90.1)	0.786
Unclear runs	7 (8.6)	8 (9.9)	
Image segment analysis			
Clear image segment	2792 (97.1)	2751 (96.5)	0.160
Suboptimal image segment	83 (2.9)	101 (3.5)	
Blood-flushing capability	0.951 (0.947–0.953)	0.950 (0.948–0.952)	0.125
Number of pullback attempt (per patient)	2.12 ± 1.01	2.42 ± 1.18	0.204
Amount of injected flushing media (mL per pullback)			
Mean with standard deviation	11.19 ± 0.77	14.84 ± 0.97	< 0.001
Median with lowest and highest deciles	11.5 (10.0, 12.0)	15.0 (13.8, 16.0)	< 0.001
Amount of injected flushing media (mL per patient)			
Mean with standard deviation	23.8 ± 11.7	36.1 ± 18.2	< 0.001
Median with lowest and highest deciles	23.0 (10.0, 41.4)	30.0 (15.0, 63.8)	< 0.001

Data are expressed as n (%), mean ± standard deviation, median (interquartile range) or median (lowest, highest deciles). *OCT* optical coherence tomography.

**Figure 4:**
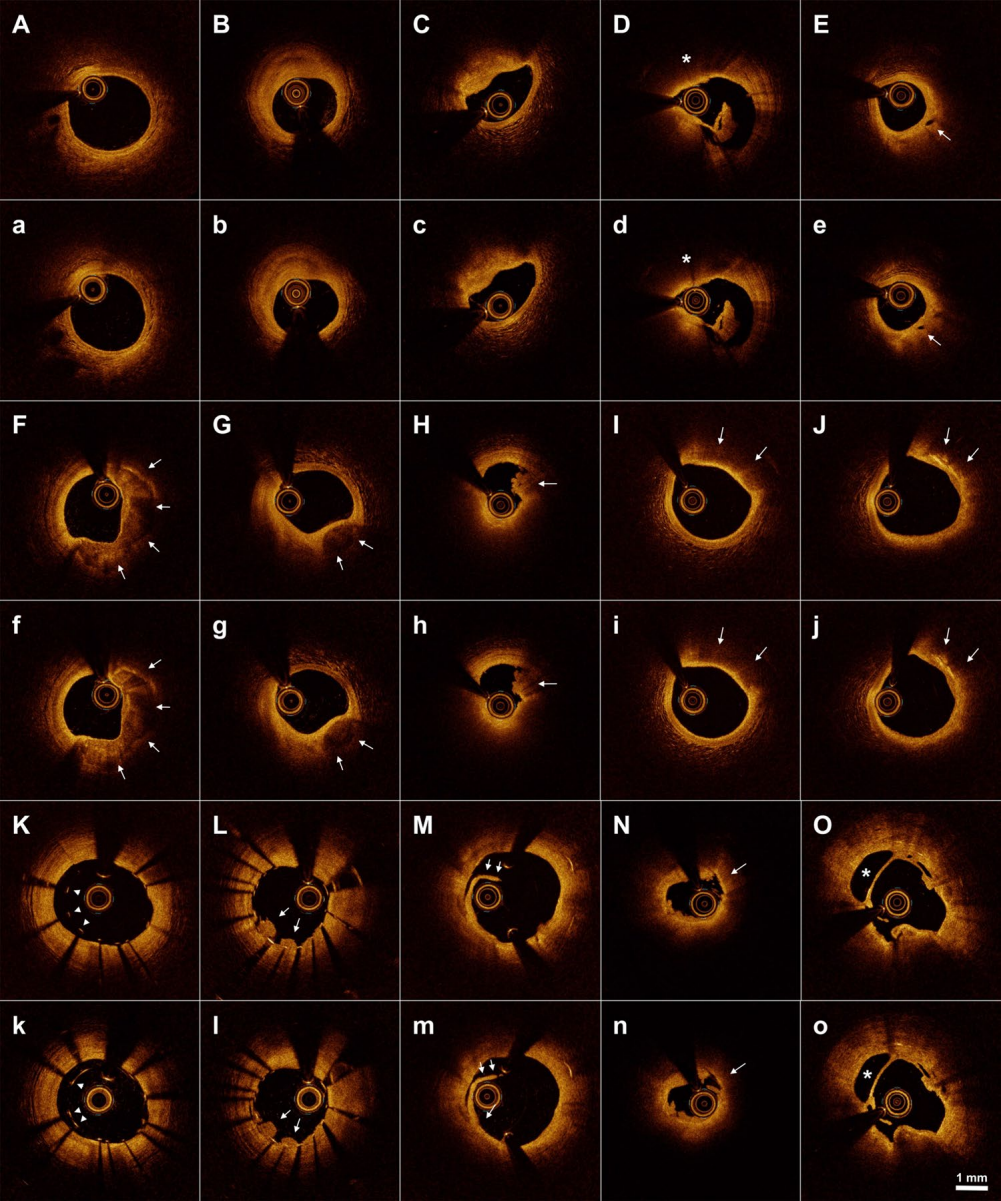
Representative OCT images of key pathological characteristics: contrast versus pentastarch. Representative cross-sectional images of the coronary atherosclerotic plaques and adverse peri-procedural findings are displayed. Comparative paired images were obtained using iodine contrast (upper panel) and pentastarch (lower panel). (**A**,**a**) normal vessel; (**B**,**b**) fibrous plaque; (**C**,**c**) loose fibrous plaque; (**D**,**d**) thin cap fibroatheroma; (**E**,**e**) vasa vasorum; (**F**,**f**) superficial eccentric calcification; (**G**,**g**) deep calcified nodule; (**H**,**h**) white thrombus; (**I**,**i**) superficial macrophage accumulation; (**J**,**j**) cholesterol crystals; (**K**,**k**) stent strut malapposition; (**L**,**l**) in-stent tissue protrusion; (**M**,**m**) intimal dissection flap; (**N**,**n**) plaque rupture; (**O**,**o**) false lumen of coronary artery dissection; white arrow, arrowhead, or asterisk indicate the main imaging characteristics. *OCT* optical coherence tomography.

Subsequently, we identified pathological OCT images of coronary atherosclerotic plaques and procedure-related adverse findings from the paired OCT pullbacks. Both image pairs performed well to precisely characterize various plaque subtypes, including fibrous, loose fibrous, calcified, and lipid-rich plaques, and to clearly visualize key pathological features of macrophage accumulation, cholesterol crystals, intimal dissection flaps, and stent-related adverse findings^2^ (Fig. 4). During post-PCI imaging, overall image quality and blood-flushing status were satisfactory to guide stent optimization in both groups of iodine contrast and pentastarch (Supplementary Video S1). Finally, we adopted a customized three-dimensional (3D)-rendered reconstruction algorithm, and both OCT pairs intuitively visualized the coronary artery microstructure and stent strut geometry of the native and stented vessels (Supplementary Videos S2 and S3).

### Comparison of quantitative morphometric measurements

**Figure 5:**
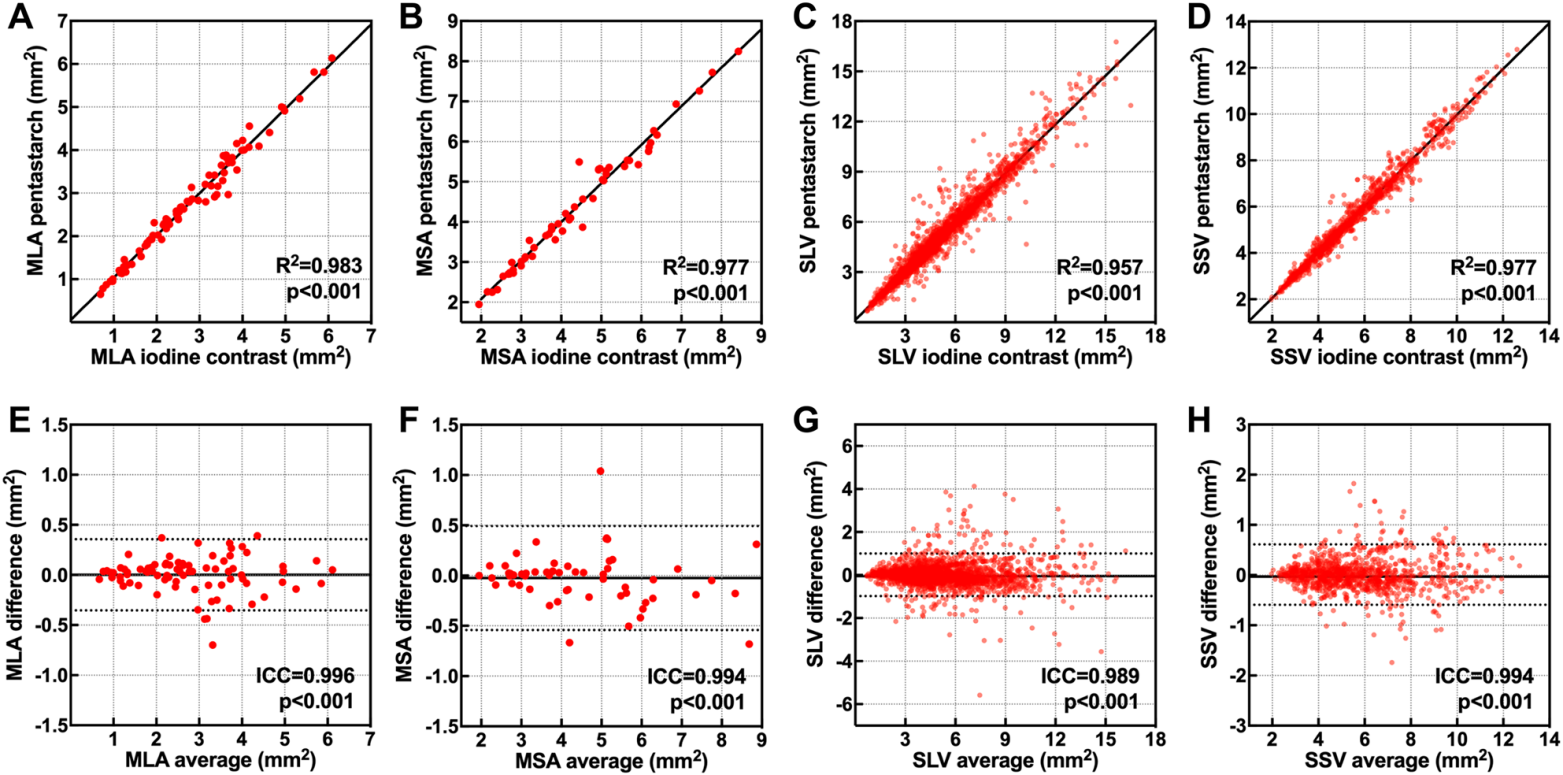
Comparison of quantitative morphometric measurements. Inter-measurement agreement of iodine contrast and pentastarch flushed images for the 2D- and 3D-morphometric measurements are displayed. (**A**,**E**) MLA; (**B**,**F**) MSA; (**C**,**G**) SLV; (**D**,**H**) SSV. *2D* two-dimensional, *3D* three-dimensional, *MLA* minimal lumen area, *MSA* minimal stent area, *SLV* segment lumen volume, *SSV* segment stent volume.

A comparison of quantitative morphometric measurements is presented in Supplementary Table S2. Both two-dimensional (2D)- (minimal lumen area [MLA] 2.86 ± 1.40 vs. 2.87 ± 1.39 mm^2^, p = 0.955; minimal stent area [MSA] 4.55 ± 1.72 vs. 4.52 ± 1.67 mm^2^; p = 0.483) and 3D-measurements (segment lumen volume [SLV] 4.95 ± 2.43 vs. 4.96 ± 2.42 mm^3^, p = 0.250; segment stent volume [SSV] 5.62 ± 2.02 vs. 5.63 ± 2.02 mm^3^; p = 0.344) showed no significant difference between the iodine contrast- and pentastarch-flushed images. Both 2D-measurements of MLA (R^2^ = 0.983; p < 0.001; Fig. 5A) and MSA (R^2^ = 0.977; p < 0.001; Fig. 5B) correlated well, and 3D-volumetric measurements of SLV (R^2^ = 0.957; p < 0.001; Fig. 5C) and SSV (R^2^ = 0.977; p < 0.001; Fig. 5D) highly correlated with each other in the paired image segments. Inter-measurement variability for the quantitative 2D- and 3D-morphometric measurements was considered excellent, with intra-class-coefficient (ICC) values ≥ 0.989 (p < 0.001; Fig. 5E–H).

### Clinical safety endpoints

**Figure 6:**
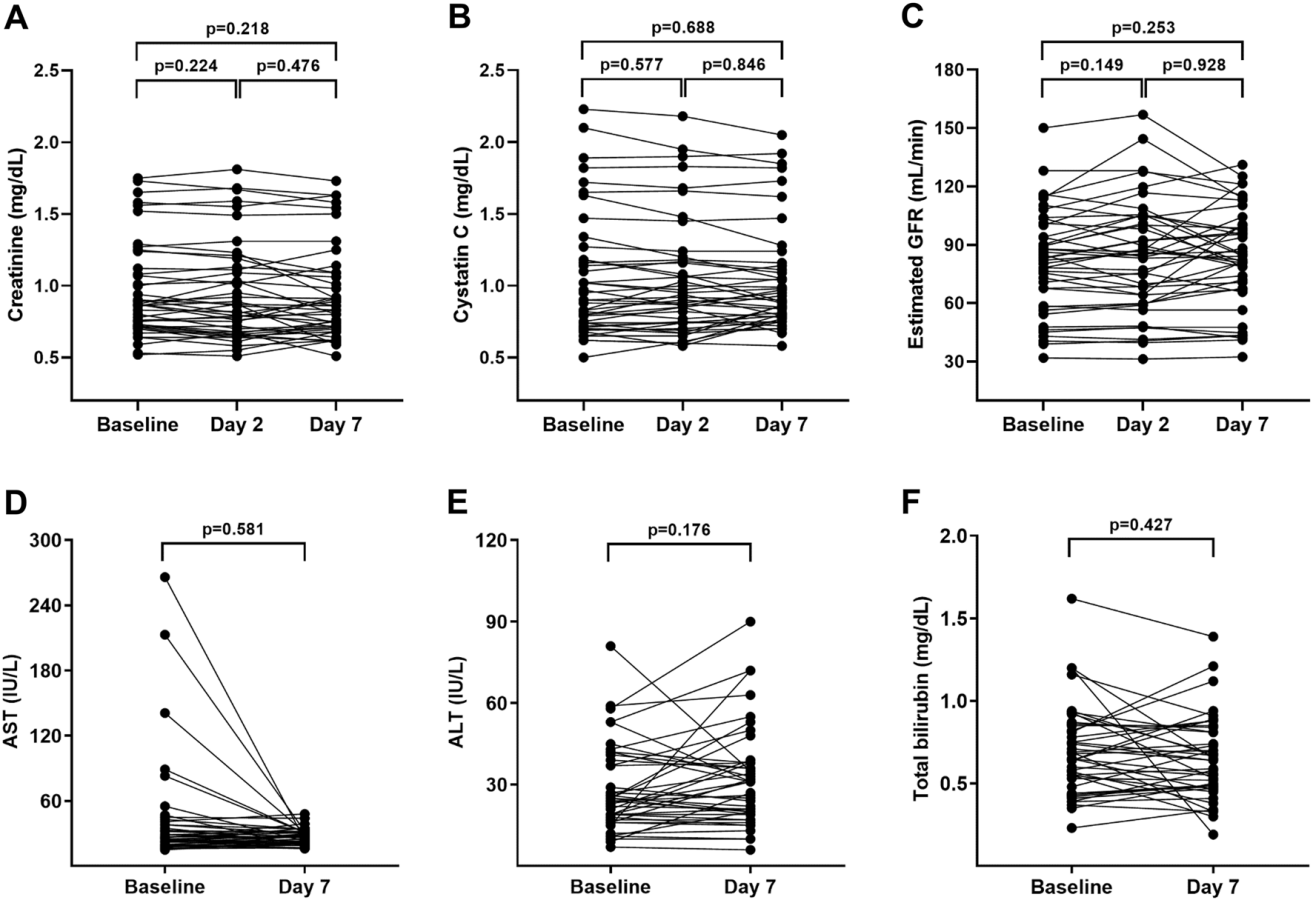
Peri-procedural change of key biosafety results. Baseline and follow-up assessment results of renal (**A**–**C**) and liver (**D–F**) functions are displayed. *ALT* alanine transaminase, *AST* aspartate transaminase, *GFR* glomerular filtration rate.

Peri-procedural changes in renal and liver function after OCT imaging with pentastarch are displayed in Fig. 6, Supplementary Tables S3 and S4. Renal function, as assessed by serum creatinine (baseline vs. day 7, 0.87 [0.72–1.12] vs. 0.83 [0.71–1.09]; p = 0.218), cystatin C (0.90 [0.72–1.18] vs. 0.90 [0.79–1.14]; p = 0.688), and estimated GFR (eGFR; 83.0 [58.5–100.0] vs. 84.1 [67.5–100.0]; p = 0.253), remained stable over seven days after the index procedure. No significant deterioration in renal function was observed at the 48–72-hour time point, and none of the patients experienced CIN. Liver function also showed no significant changes during the seven days follow-up. All patients (n = 43) safely underwent intravascular OCT imaging using both iodine contrast and pentastarch without resulting in clinically relevant adverse events, such as procedural mortality, life-threatening arrhythmia, anaphylactic reaction, heart failure, or hemodynamic instability (Supplementary Table S5).

## Discussion

To the best of our knowledge, this is the first-in-the-field prospective comparison to demonstrate the feasibility and safety of non-contrast OCT imaging using pentastarch. The pentastarch solution provided sufficient OCT image quality that is comparable to conventional iodine contrast, thus enabled detailed microstructural assessment and guaranteed highly correlative 2D- and 3D-morphometric measurements. The blood-flushing capability of pentastarch, as assessed by an automated pixel-based comparison algorithm, was equivalent to that of iodine contrast. The overall image quality of OCT pullbacks using pentastarch was excellent for intuitively visualizing the intravascular lumen geometry by customized 3D-rendered reconstruction and was successful for facilitating stent optimization. Intracoronary administration of pentastarch during OCT imaging was clinically safe without resulting any significant clinical adverse events. Key biosafety markers, including renal and liver function tests, remained stable up to seven days after the index procedure. Collectively, our study results provide robust clinical evidence for pentastarch as an alternative blood-flushing medium for intracoronary OCT imaging.

The clinical utilization of contemporary OCT is frequently hampered by the use of iodine contrast for blood elimination. The additional amount of iodine contrast required for OCT image acquisition is undesirable and potentially increases the risk of CIN, especially in patients with impaired renal function^4,5^. Therefore, various clinical efforts have been made to find alternative flushing media that can generate comparable image quality and minimize renal damage. Previous studies have suggested that fluid solutions, including low-molecular-weight dextran sulfate, diluted iodine contrast, or even normal saline could be used as alternative blood-flushing media for intracoronary OCT imaging^8,15–21^. Few studies have shown that low-molecular-weight dextran can provide similar image quality and measurement indices during OCT imaging compared to iodine contrast^15,16^. Although dextran has the advantages of low cost and relative clinical safety in small-volume administration, it could potentially result in adverse clinical events such as anaphylactic reactions, dose-dependent coagulopathy, and renal dysfunction^9,22,23^. Various combinations of diluted iodine contrast have been investigated and reported to provide similar image quality to pure iodine contrast in OCT imaging^19,21^. The use of diluted iodine contrast could modestly reduce the obligatory contrast load; however, the cumulative dose inevitably increases by repetitive imaging, and the optimal dilution ratio needs to be standardized for broader application. Recently, small sample-sized studies have examined the feasibility of normal saline for OCT image acquisition^17,18,20^. Although saline flushing provided grossly acceptable image quality for clinical utilization, blood-flushing capability varied by individual studies, and low viscosity of saline inherently increased the rate of incomplete flushing compared to iodine contrast^17,18^. In addition, despite refractive index correction, saline flushed-OCT images can overestimate or underestimate in vivo quantitative measurements, thus hampering their universal application^17^. Considering these issues raised from previously suggested alternative flushing media, different fluid solutions should be investigated as acceptable substitutes to conventional iodine contrast.

Optimal flushing media for intravascular OCT should have sufficient blood-flushing capability for image acquisition and should guarantee accurate morphometric measurements. At body temperature, pentastarch (4.25 cP at 37 °C) has one-third to half the strength viscosity of iodine contrast (Iodixanol, 11.4 cP at 37 °C), which is strong enough to eliminate red blood cells^14,24^. The overall image quality, as assessed by the number of CIS and clear runs, was similar between OCT pullbacks using pentastarch and iodine contrast. To overcome the inherent limitation of the intermittently assessed qualitative index (e.g., CIS), we newly constructed a sophisticated automated customized pixel-based comparison algorithm that could directly measure blood-flushing status from each cross-sectional image segment. Our pixel-based quantitative assessment confirmed that pentastarch has an excellent blood-flushing capability comparable to that of iodine contrast for OCT imaging. Blood elimination by pentastarch flushing clearly identified various plaque subtypes and peri-procedural adverse findings to successfully guide treatment optimization. In morphometric analysis, pentastarch-flushed images enabled the accurate assessment of 2D-cross-sectional measurements including MLA and MSA. Furthermore, we conducted a volumetric correlative analysis to examine measurement agreement across the overall imaged vessel segments. Corresponding 1 mm-length cylindrical segments were matched with each other, and 3D-volumetric measurements of SLV and SSV were highly correlated between the paired images of iodine contrast and pentastarch. In summary, our detailed morphometric analysis provides robust evidence to support the feasibility of pentastarch for clinical OCT imaging and strongly suggests its potential as an acceptable alternative to iodine contrast.

Pentastarch has been widely used as a plasma volume expander in various clinical settings that require volume replacement therapy^9,10,25^. However, in critically ill patients with severe sepsis and septic shock, large-volume administration of HES has been reported to increase the risk of renal impairment^25–27^. Although small dose administration of HES solution is considered to be safe^9,12,13^, regarding the conflicting issues on HES solution in septic patients, clinical safety evidence should be provided for intracoronary injection of pentastarch. Therefore, in this first-in-the-field explorative study, we thoroughly assessed the clinical safety of pentastarch for intracoronary administration. Peri-procedural renal function was comprehensively assessed using various measurement indices, including serum creatinine, cystatin C, and eGFR. Both renal and liver functions remained stable over the seven days follow-up and all patients safely underwent intracoronary injection of pentastarch without experiencing any complicating peri-procedural events. Furthermore, in the present study, low-volume administration (< 100 mL) of pentastarch was safely conducted in patients with a reduced left ventricular ejection fraction (< 50%) and ACS without resulting in significant adverse events such as volume retention, coagulopathy, or anaphylactic reaction. These findings suggest that pentastarch could be safely used as an alternative flushing solution for OCT imaging in various clinical settings of coronary artery disease (CAD).

In addition to its proven clinical safety for intracoronary administration, pentastarch also offers significant advantages as an alternative flushing media for OCT imaging. It is noteworthy that various HES solutions are widely available worldwide in numerous countries. Different generic brands of 10% HES (250 kDa/0.45) prepared in 0.9% NaCl are expected to demonstrate equivalent characteristics with pentastarch in terms of viscosity, composition, and optical index for intracoronary OCT imaging. The newer generation HES solutions (6% HES 130 kDa/0.4) are also currently available from the global market, however, their lower viscosity profile (1.74 cP at 37 °C) poses challenges in blood clearance and has been reported to substantially compromise image quality during OCT imaging^14,28^. Given its global availability and sufficient viscosity profile, pentastarch could be considered a suitable option for non-contrast OCT imaging among various HES solutions. Another potential advantage of pentastarch is its favorable cost-effectiveness compared to conventional iodine contrast. While costing variations may exist across different countries, the estimated cost per 100 mL volume of pentastarch is approximately 10 times lower compared to that of iodine contrast.

Intracoronary OCT allows tomographic visualization of coronary microstructures and greatly enhances our understanding of CAD^1,2^. Recently, intracoronary OCT is evolving into a multi-modal imaging technology by combining complementary fluorescence signals which simultaneously characterize multiple biochemical composition of atherosclerotic plaques^29,30^. The use of conventional iodine contrast as a blood-flushing medium is often restricted for this multi-modal OCT imaging since the considerable absorption property of the iodine contrast, especially in the ultraviolet-blue spectrum, might affect the diagnostic performance of fluorescence imaging^31^. Hybrid OCT technology combined with fluorescence lifetime imaging (FLIm), which utilizes ultraviolet light as an excitation source, is mandatory for contrast-free blood elimination with an optically transparent solution during image acquisition^30,32^. Pentastarch, which ensures excellent blood elimination with high optical transparency and proven biosafety, could safely replace iodine contrast and could be used as the main flushing solution for next-generation multi-modal OCT imaging including OCT-FLIm (NCT04835467).

Our study had several limitations. First, this was a single-center, non-randomized, observational study with a relatively small sample size. We implemented a sophisticated pixel-based comparison algorithm to support the robustness of our key findings; however, these should be further validated in a large-scale study with a greater sample size. Second, OCT imaging was performed by manually injecting the flushing media. Although the overall image quality was excellent by manual injection of pentastarch at a flow rate of 4.0 mL/s, personal variation could be present across the different operators. Further validation with automated power injector system should be conducted to standardize injection regimen and to enable broader clinical application. Third, the level of renal dysfunction was mostly confined to a mild degree of stage 3 CKD in our study due to the exclusion of individuals with a serum creatinine level greater than 2.0 mg/dL. Although the present findings could serve as a fundamental basis for expanding the application of pentastarch for non-contrast OCT imaging, additional evidence should be provided for those with more severe forms of renal dysfunction. Fourth, the administered amount of pentastarch was relatively larger than that of iodine contrast for OCT image acquisition. An average 15 mL of pentastarch was required for a single OCT pullback; however, intracoronary injection of average 36.1 ± 18.2 mL of pentastarch per patient for multiple image acquisition in addition to iodine contrast was found to be safe without resulting in clinical adverse events. Fifth, the present study was limited to separately assessing the safety profile of iodine contrast versus pentastarch for OCT image acquisition. The clinical safety profile shown in our study should be interpreted with caution by considering the combined use of iodine contrast and pentastarch. As the first-in-the-field investigation to demonstrate feasibility of pentastarch for non-contrast OCT imaging, our findings can offer supporting evidence to guide designing a large-scale prospective comparative study. In the future, patient-level comparative studies should explore the biological safety of administering higher volumes of pentastarch compared to iodine contrast in various clinical scenarios involving complex PCI or multi-vessel OCT imaging of non-culprit lesions.

Compared to iodine contrast, intracoronary OCT using pentastarch is technically feasible, clinically safe, and does not affect the overall image quality, blood-flushing capability, and morphometric measurements. Non-contrast imaging with pentastarch could be a promising strategy for patients at high risk of renal impairment by substantially reducing the obligatory iodine contrast load. While further large-scale studies employing a patient-level comparison are needed, the present findings highlight pentastarch as an alternative flushing medium for non-contrast OCT imaging.

## Methods

### Study design and population

This was a prospective, single-center, open-label, comparative study conducted in the Cardiovascular Center at Korea University Guro Hospital (KUGH; Seoul, Republic of Korea). Patients undergoing OCT-guided PCI for CSA or ACS were prospectively enrolled between March 2021 and January 2022. The flow chart of the study is shown in Fig. 1. The study protocol complied with the Declaration of Helsinki and was approved by the Ethics Committee and Institutional Review Board at KUGH (2023GR0036). Written informed consent was obtained from all participants prior to enrollment. Key exclusion criteria for study enrollment were as follows: (1) hemodynamic instability, (2) renal dysfunction (serum creatinine > 2.0 mg/dL), (3) overt pulmonary congestion (Killip class III or IV), and (4) unfavorable vessel anatomy for intracoronary OCT imaging (e.g., highly tortuous vessel, vessel diameter < 2.0 mm or > 5.0 mm).

### Clinical data collection and patient management

Demographic features and underlying cardiovascular risk factors were recorded through detailed patient interviews. Baseline laboratory data were obtained at the time of the initial admission for PCI. The diagnosis of ACS was based on clinically relevant angina symptoms, increased cardiac biomarkers and typical changes on 12-lead electrocardiography. The presence of CKD and heart failure was defined as estimated glomerular filtration rate less than 60 mL/min by the Modification of Diet in Renal Disease equation^33^ and left ventricular ejection fraction < 50%, respectively. Detailed procedural strategies and post-PCI medications were determined at the discretion of the attending cardiologist in accordance with contemporary practice guidelines^34,35^.

### OCT image acquisition

OCT imaging was performed in all patients using either a 6-Fr or 7-Fr guiding catheter via radial or femoral approach. All patients received an intravenous bolus injection of 5000 IU heparin. An intracoronary injection of 100–200 μg nitroglycerin was administered to prevent catheter-induced coronary spasm. OCT imaging was performed using the ILUMIEN^®^ OCT imaging system and Dragonfly OPTIS^®^ imaging catheter (Abbot Vascular, Santa Clara, CA, USA). The contemporary intravascular OCT system provides images at a frame rate of 100 images/s, with an axial resolution of ~ 20 μm and a lateral resolution of 25–60 μm. All OCT images were acquired at a pullback speed of 20 mm/s, a pullback length of 54 mm, and a frame spacing of 0.2 mm with manual injection of blood-flushing media, warmed at 37 °C. OCT pullbacks were repeated from the same imaging segment by alternatively injecting iodine contrast (Visipaque^®^, Iodixanol, GE Healthcare, Chicago, IL, USA) and pentastarch (Jeil Pentastarch^®^, 10% HES 250 kDa/0.45; Jeil Pharmaceutical, Seoul, Republic of Korea) by cardiac interventionists specializing in OCT imaging. While a 12 mL control syringe was used for manual injection of iodine contrast, a 30 mL Luer lock syringe was utilized for pentastarch to enable higher volume administration beyond the limit of the 12 mL control syringe. The estimated manual injection rate of pentastarch was 4.0 mL/s, administered over a maximum duration of four seconds. Native vessel imaging was performed before PCI in available cases of culprit and/or non-culprit lesions, and all stented lesions were imaged after PCI to optimize stent implantation. All recorded images were stored externally for offline customized quantitative analysis.

### Automated customized pixel-based algorithm for OCT image analysis

All OCT images were analyzed using a customized image-processing toolkit written in MATLAB (R2019a; MathWorks, Natick, MA, USA). Figure 2 shows the overall scheme of the comparative OCT image analysis. An automated customized pixel-based algorithm, developed by our research group^36^, dedicated to lumen contour delineation and stent strut detection was adopted for objective and quantitative comparative analysis. The dominant edges in the depth direction and bright reflections with a dark tail were identified to delineate the lumen contour and to detect the stent struts, respectively. This methodology can be implemented using gradient images along the depth direction. Further refinement to eliminate false positive stent struts was achieved using a neural-network-based classifier. The stent contour was delineated by connecting the abluminal surfaces of the detected struts as a smooth, closed-loop using cubic smoothing spline interpolation^37^.

Analysis of the corresponding image segment was ascertained using fiduciary landmarks, such as side branches and implanted stents, between the paired OCT pullbacks of iodine contrast and pentastarch. OCT image quality was assessed by the cumulative number of CIS as previously described^15^. CIS was defined as a 1-mm longitudinal segment containing at least 50% clear images in which the arterial structure was clearly visualized along a continuous arc > 270°^38^. Individual OCT pullbacks were defined as clear runs if at least 90% of the 1-mm longitudinal segments were classified as CIS. Blood-flushing capability was assessed by calculating the number of residual blood pixels within the vessel lumen after excluding OCT catheter sheath and guide-wire artifacts. Considering that blood remnant is presented as “bright” debris floating inside the vessel lumen, in this study, residual blood pixels were defined as “bright” pixels with value greater than a predetermined background threshold. Accordingly, blood-flushing capability can be quantified at individual cross-sections as follows:

$${\text{Blood}}\,{\text{flushing}}\,{\text{capability}} = 1 - \frac{{{\text{number}}\,{\text{of}}\,{\text{bright}}\,{\text{pixels}}\,{\text{within}}\,{\text{the}}\,{\text{vessel}}\,{\text{lumen}}}}{{{\text{lumen}}\,{\text{area}} - {\text{area}}\,{\text{of}}\,{\text{OCT}}\,{\text{catheter}}\,{\text{and}}\,{\text{guidewire}}}}.$$

In terms of quantitative 2D-morphometric analysis, the lumen and stent areas were measured at each cross-sectional frame. The MLA and MSA were identified from the paired OCT pullbacks. The 3D-volumetric measurements of SLV and SSV were derived from the 1-mm longitudinal segments of native and stented vessels. Finally, the paired OCT images were 3D-reconstructed using the OsiriX software (The OsiriX Foundation, Geneva, Switzerland) for intuitive visualization of coronary microstructures and stent strut geometry. Since the refractive properties of pentastarch differ from those of iodine contrast (measured refractive index at the OCT wavelength, pentastarch vs. iodine contrast = 1.3433 vs. 1.4421), morphometric measurements from the pentastarch group were adjusted by refractive index correction to avoid underestimation.

### Clinical safety endpoints

Clinical safety endpoints, including renal and liver function tests, were assessed at the time of admission and followed up for seven days after the index procedure. The primary safety endpoint was the temporal change in serum creatinine over seven days after OCT imaging with pentastarch. The secondary safety endpoint was changes in serum cystatin C and eGFR over the seven days follow-up. CIN was defined as an increase in serum creatinine > 0.5 mg/dL or > 25% within 72 hours^39^. Other peri-procedural and patient-oriented clinical adverse events were monitored up to 30 days.

### Statistical analysis

Sample size calculation determined that a minimum of 44 OCT pullback pairs would yield 90% power to detect an effect size of 0.50 at a significance level of 5% in the paired assessment of blood-flushing capability. Our sample size of 81 OCT pullback pairs from 43 patients had sufficient statistical power to quantitatively validate blood-flushing capability. Data were expressed as mean and standard deviation or median and interquartile range (IQR) for continuous variables and frequency (percentage) for categorical variables. Differences between groups were analyzed using Student’s t-test or Mann–Whitney U-test for continuous variables and Pearson’s chi-square or Fisher’s exact test for categorical variables. Differences in paired measurements were examined using paired t-test or non-parametric Wilcoxon signed-rank test. Correlations between the paired OCT morphometric measurements were examined using Pearson’s correlation coefficient. Inter-measurement variability was assessed using the ICC and visualized by Bland–Altman plots. All analyses were two-tailed, and significant differences were defined as p < 0.05. Statistical analyses were performed using Statistical Package for the Social Sciences software (version 20.0; SPSS-PC Inc., Chicago, IL, USA) and Prism (version 9.4.1; GraphPad Software Inc., San Diego, CA, USA).

### Supplementary Information

Supplementary Information.

Supplementary Video 1.

Supplementary Video 2.

Supplementary Video 3.

## Data Availability

The corresponding author has full access to the study data, and anonymized data will be available upon request from a qualified researcher.
