# Supplementary material for: Feasibility and safety of non-contrast optical coherence tomography imaging using hydroxyethyl starch in coronary arteries
Source: Sci Rep. 2023 Aug 24;13:13818. doi: 10.1038/s41598-023-40363-7 (PMC10449772; doi:10.1038/s41598-023-40363-7)
Supplement: Supplementary file 1 — Supplementary Information. [file 41598_2023_40363_MOESM1_ESM.docx]

**Supplementary Data**

**Feasibility and Safety of Non-contrast Optical Coherence Tomography Imaging using Hydroxyethyl Starch in Coronary Arteries**

Dong Oh Kang, MD, PhD^a,b,*^, Hyeong Soo Nam, PhD^c,*^, Sunwon Kim, MD, PhD^a,d^, Hongki Yoo, PhD^c,†^ and Jin Won Kim, MD, PhD^a,b,†^

^a^Multimodal Imaging and Theranostic Lab, Cardiovascular Center, Korea University Guro Hospital, Seoul, Republic of Korea

^b^Cardiovascular Center, Korea University Guro Hospital, Seoul, Republic of Korea
^c^Department of Mechanical Engineering, Korea Advanced Institute of Science and Technology, Daejeon, Republic of Korea
^d^Cardiovascular Center, Korea University Ansan Hospital, Ansan, Republic of Korea

^*^These authors contributed equally to this work.
^†^These authors jointly supervised this work.

**Supplementary Tables**

**Supplementary Table S1.** **Comparative analysis of OCT images obtained using the 6-Fr and 7-Fr guiding catheters with pentastarch solution**

|  | **6-Fr catheter** | **7-Fr catheter** | **P-value** |
| --- | --- | --- | --- |
| **Baseline characteristics** |  |  |  |
| LVEF (%) | 60.0 (51.5–60.0) | 48.0 (44.0–60.0) | 0.045 |
| Serum creatinine (mg/dL) | 0.80 (0.70–0.95) | 0.91 (0.76–1.53) | 0.061 |
| Cystatin C (mg/dL) | 0.79 (0.69–1.03) | 1.02 (0.81–1.64) | 0.002 |
| **Pentastarch** |  |  |  |
| Pixel-based blood-flushing capability | 0.950 (0.949–0.951) | 0.951 (0.948-0.952) | 0.663 |
| Amount of injected volume (mL per pullback) | 14.33 ± 0.66 | 15.32 ± 0.99 | <0.001 |

Data are expressed as mean ± standard deviation or median (interquartile range). LVEF, left ventricular ejection fraction.

**Supplementary Table S2. Quantitative morphometric measurements and inter-measurement variability assessment**

|  | **Quantitative measurements** | | | **Correlative analysis** | | **Inter-measurement variability** | |
| --- | --- | --- | --- | --- | --- | --- | --- |
|  | **Iodine contrast** | **Pentastarch** | **P-value** | **R^2^** | **P-value** | **ICC (95% CI)** | **P-value** |
| 2D-measurements |  |  |  |  |  |  |  |
| MLA (mm^2^) – 81 pairs | 2.86 ±1.40 | 2.87 ±1.39 | 0.955 | 0.983 | <0.001 | 0.996 (0.993–0.997) | <0.001 |
| MSA (mm^2^) – 56 pairs | 4.55 ± 1.72 | 4.52 ± 1.67 | 0.483 | 0.977 | <0.001 | 0.994 (0.990–0.996) | <0.001 |
| 3D-measurement |  |  |  |  |  |  |  |
| SLV (mm^3^) – 2960 pairs | 4.95 ± 2.43 | 4.96 ± 2.42 | 0.250 | 0.957 | <0.001 | 0.989 (0.988–0.990) | <0.001 |
| SSV (mm^3^) – 1163 pairs | 5.62 ± 2.02 | 5.63 ± 2.02 | 0.344 | 0.977 | <0.001 | 0.994 (0.993–0.995) | <0.001 |

Data are expressed as the mean ± standard deviation or median (interquartile range). 2D, two-dimensional; 3D, three-dimensional; CI, confidence interval; ICC, intra-class correlation coefficient; MLA, minimal luminal area; MSA, minimal stent area; SLV, segment lumen volume SSV, segment stent volume

**Supplementary Table S3.** **Peri-procedural changes of renal function**

|  | **Baseline** | **Day 2** | **Day 7** |
| --- | --- | --- | --- |
| **Renal function test** |  |  |  |
| Serum creatinine (mg/dL) | 0.87 (0.72–1.12) | 0.86 (0.68–1.13) | 0.83 (0.71–1.09) |
| Cystatin C (mg/dL) | 0.90 (0.72–1.18) | 0.92 (0.74–1.18) | 0.90 (0.79–1.14) |
| eGFR (mL/min) | 83.0 (58.5–100.0) | 84.3 (59.8–103.8) | 84.1 (67.5–100.0) |
| **P-value** | **Baseline vs. Day 2** | **Baseline vs. Day 7** | **Day 2 vs. Day 7** |
| Serum creatinine (mg/dL) | 0.224 | 0.218 | 0.476 |
| Cystatin C (mg/dL) | 0.577 | 0.688 | 0.846 |
| eGFR (mL/min) | 0.149 | 0.253 | 0.928 |

Data are expressed as the median (interquartile range). eGFR, estimated glomerular filtration rate

**Supplementary Table S4. Peri-procedural changes of liver function**

|  | **Baseline** | **Day 7** | **P-value** |
| --- | --- | --- | --- |
| **Liver function test** |  |  |  |
| AST (IU/L) | 25.0 (19.0-38.0) | 27.0 (22.0-31.0) | 0.581 |
| ALT (IU/L) | 24.0 (17.0-39.0) | 31.0 (19.0-38.0) | 0.176 |
| Total bilirubin (mg/dL) | 0.65 (0.48-0.85) | 0.64 (0.48-0.81) | 0.427 |

Data are expressed as the median (interquartile range). ALT, alanine transaminase; AST, aspartate transaminase

**Supplementary Table S5. Clinical safety endpoints**

|  | **n (%)** |
| --- | --- |
| Procedural mortality | 0 (0.0) |
| Acute kidney injury | 0 (0.0) |
| Contrast induced nephropathy^*^ | 0 (0.0) |
| Life-threatening arrhythmia | 0 (0.0) |
| Hemodynamic instability | 0 (0.0) |
| Heart failure | 0 (0.0) |
| Anaphylaxis reaction | 0 (0.0) |

Data are expressed as n (%). ^*^Contrast-induced nephropathy was defined as an increase in serum creatinine > 0.5 mg/dL or > 25% within 72 hours.

**Supplementary Figure**

**Supplementary Figure S1.** **Distribution of CIS in the pullbacks of iodine contrast and pentastarch.**

The distribution of CIS per pullback in the groups of iodine contrast and pentastarch is displayed. CIS, clear image segment; PB, pullback


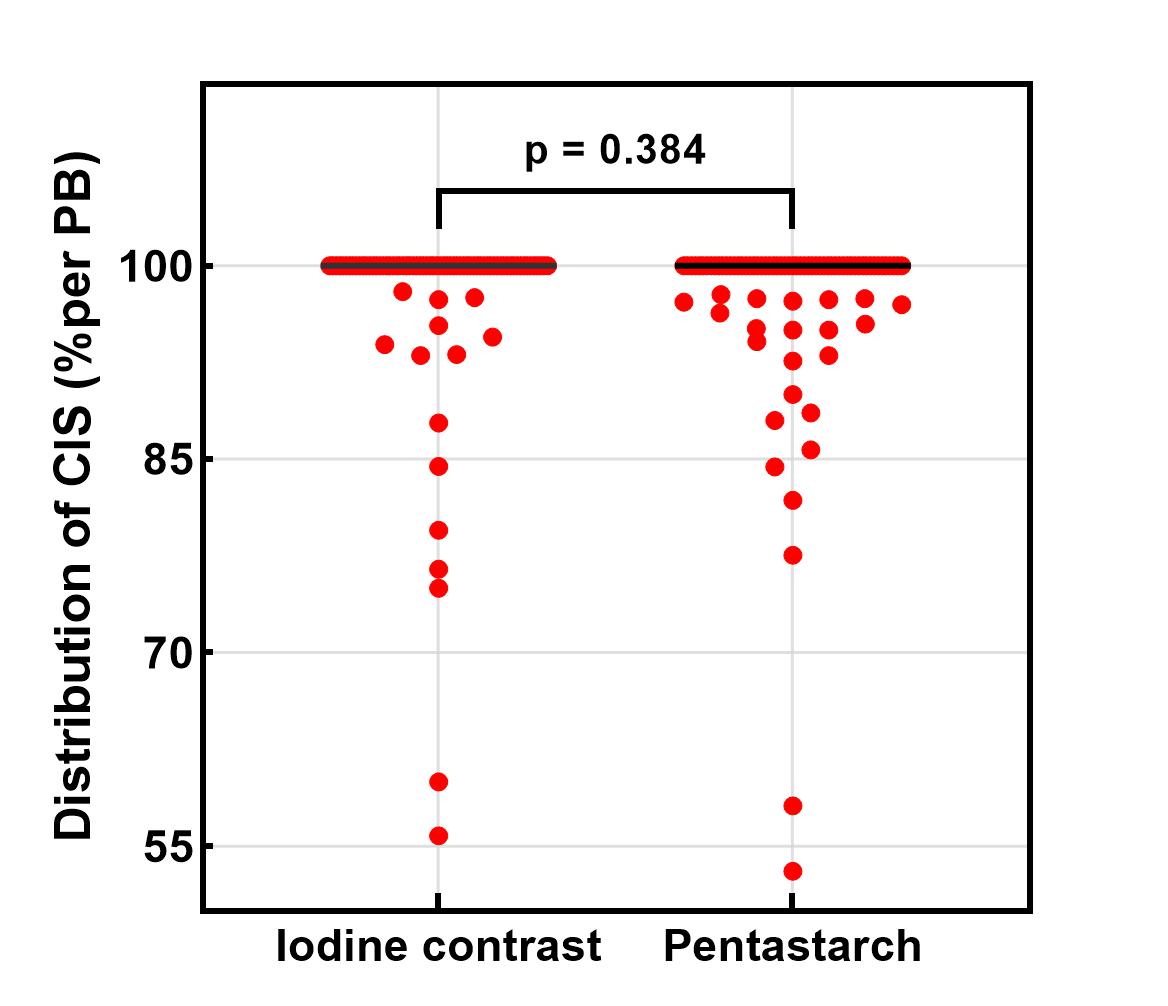


**Supplementary Video Legend**

**Supplementary Video S1.** **Representative clinical OCT image of stented vessel using iodine contrast and pentastarch**OCT, optical coherence tomography

**Supplementary Video S2. Customized 3D-rendered reconstruction and fly-through OCT images of native vessel: iodine contrast versus pentastarch.**

An intracoronary OCT image of a native vessel obtained from a 71-year old male patient presented with non-STEMI. ^*^acute marginal branch; 3D, three-dimensional; OCT, optical coherence tomography; PCT, percutaneous coronary intervention; STEMI, ST-elevation myocardial infarction.

**Supplementary Video S3. Customized 3D-rendered reconstruction and fly-through OCT images of stented vessel: iodine contrast versus pentastarch**.

An intracoronary OCT image of a stented vessel obtained from a 48-year old male patient presented with stable angina. ^*^septal branch; 3D, three-dimensional; DES, drug-eluting stent; OCT, optical coherence tomography
